# Family-wide analysis of integrin structures predicted by AlphaFold2

**DOI:** 10.1101/2023.05.02.539023

**Published:** 2023-05-02

**Authors:** Heng Zhang, Daniel S. Zhu, Jieqing Zhu

**Affiliations:** 1 Versiti Blood Research Institute, Milwaukee, WI, USA.; 2 Department of Biochemistry, Medical College of Wisconsin, Milwaukee, WI, USA.; 3 D.Z. is a student intern from Brookfield Central High School, Brookfield, WI, USA

## Abstract

Recent advances in highly accurate protein structure prediction by AlphaFold have opened new avenues for analyzing all structures within a single protein family. In this study, we evaluated the capacity of the newly developed AlphaFold2-multimer for predicting integrin heterodimers. Integrins are heterodimeric cell surface receptors made up of a combination of 18 α and 8 β subunits, forming a family of 24 different members. Both α and β subunits contain a large extracellular domain, a short transmembrane domain, and usually a short cytoplasmic domain. Integrins play a wide range of cellular functions by recognizing diverse ligands. Structural studies in recent decades have greatly advanced our understanding of integrin biology, but high-resolution structures have only been determined for a few members of the integrin family. We studied the single-chain atomic structures of 18 α and 8 β integrins in the AlphaFold2 protein structure database. We then applied the AlphaFold2-multimer program to predict the α/β heterodimer structures of all 24 human integrins. The results show a high level of accuracy in the predicted structures for the subdomains of both α and β subunits and provide high-resolution structure information of all integrin heterodimers. Our structural analysis of the entire integrin family reveals a potentially diverse range of conformations among the 24 members and provides a useful structure database for guiding functional studies. However, our results also suggest the limitations of AlphaFold2 structure prediction and thus caution is required in the interpretation and usage of the AlphaFold2 structures.

## Introduction

**Figure 1.**
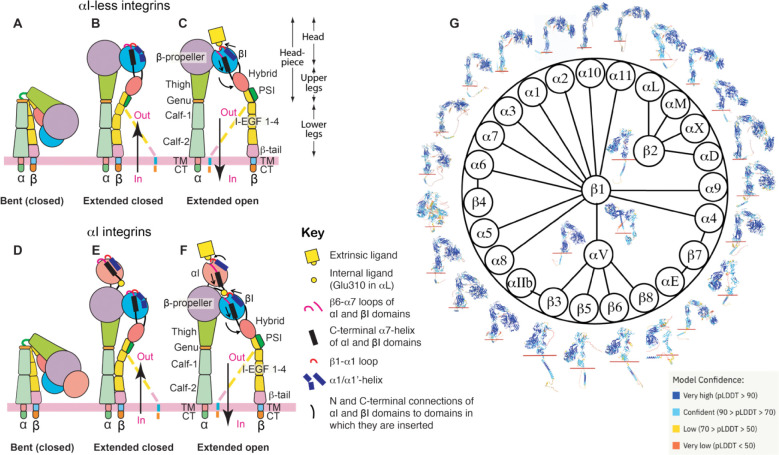
Integrin domain organization and structures predicted by AlphaFold2. (**A-F**) Integrin domain organization and conformational changes during activation. The αI-less (A-C) and αI-containing (D-F) integrins are shown separately. The global and local structural changes are indicated. The dashed lines indicate the alternative conformations of the β leg domains. (**G**) Integrin family and overall structures of α and β subunits predicted by AlphaFold2. The red line indicates the boundary between the extracellular and transmembrane (TM) domain. The structure images are color coded based on model confidence.

Integrins are cell surface receptors that recognize a variety of extracellular or cell surface ligands, allowing cells to integrate signals from both inside and outside of the cell.^1^ The human integrin family consists of 24 members, which result from the combination of 18 α and 8 β subunits (Fig. 1). The 24 α/β integrin heterodimers are either widely distributed or specifically expressed in certain cell types, thus playing common or specialized functions in cellular responses related to cell adhesion and migration. Based on ligand or cell specificity, integrins can be classified into subfamilies of RGD (Arg-Gly-Asp) receptors, collagen receptors, laminin receptors, and leukocyte-specific receptors.^1^ Integrins are key players in diseases such as thrombosis, inflammation, and cancer, making them prime targets for small molecule or antibody inhibitors.^2–4^ Since their discovery in the early 1980s, understanding the structure and function of integrins has been an area of continuous research interest.^5^

The α and β subunits of integrin are composed of multiple subdomains. The α subunit contains β-propeller, thigh, calf-1, calf-2, transmembrane (TM), and cytoplasmic tail (CT) domains (Fig. 1A–C). The β subunit contains βI, hybrid, PSI, I-EGF 1–4, β-tail, TM, and CT domains (Fig. 1A–C). A subclass of α integrins have an extra αI domain inserted into the β-propeller domain (Fig. 1D–F). For αI-less integrins, the α β-propeller and β βI domains come together to form the ligand binding site (Fig. 1A–C). For αI integrins, the αI domain is responsible for binding ligands (Fig. 1D–F). Integrin domains can also be divided into headpiece (containing head and upper legs) and lower legs (Fig. 1C). In the past few decades, structural studies of integrins have revealed a conformation-dependent activation and ligand binding mechanism, involving a transition among at least three conformational states.^6, 7^ The bent conformation with closed headpiece represents the resting state of integrin (Fig. 1A, D), while the extended closed headpiece and extended open headpiece represent the intermediate and high-affinity active states, respectively (Fig. 1B–C, E–F). The conformational transition of integrin can be initiated by the binding of intracellular activators, including talin and kindlin, to β CT, resulting in inside-out signaling, or by the binding of extracellular ligands, resulting in outside-in signaling (Fig. 1A–F).^8, 9^ The conformation-dependent activation model, however, was largely derived from structural studies of the highly-regulated β_2_ and β_3_ integrins that are primarily expressed in blood cells.^6, 7^ Since structural information for most integrin members is lacking, it remains unclear whether the current model of integrin conformational change can be applied to the entire integrin family.

Since the publication of the first high-resolution crystal structure of the α_V_β_3_ ectodomain in 2001,^10^ significant efforts have been made to determine integrin structures using various methods, including crystallography, negative-stain electron microscopy (EM), nuclear magnetic resonance (NMR), and more recently, cryogenic EM (cryo-EM). However, high-resolution structure information is still limited to a few of integrin members, including α_V_β_3_,^10–12^ α_IIb_β_3_,^13–21^ α_5_β_1_,^22–24^ α_6_β_1_,^25^ α_X_β_2_,^26, 27^ α_L_β_2_,^28^ α_M_β_2_,^29, 30^ α_V_β_6_,^31–33^ α_V_β_8_,^34–37^ and α_4_β_7_,^38^ many of which only have the fragment structures determined to date. Also, among the 24 integrins, only the α_IIb_β_3_ TM-CT heterodimer structures has been determined experimentally.^20, 39–43^ The recent breakthrough in protein structure prediction using the artificial intelligence-based AlphaFold2 program has provided a powerful tool for analyzing previously hard-to-determine protein structures with a high level of accuracy.^44^ We analyzed the predicted atomic structure models of single-chain 18 α and 8 β integrins that are available in the AlphaFold2 database (Fig. 1G). Furthermore, using the recently developed AlphaFold2-multimer program,^45^ we predicted the structures of all 24 human integrin α/β heterodimers. Our analysis of the integrin family structures revealed potential conformational diversity across its 24 members. We also identified previously unknown structural features, and created a comprehensive database of integrin structures that can guide functional and structural studies. These findings highlight the effectiveness of AlphaFold2 in predicting the structures of large, complex protein families, including integrins.

## Methods

### Databases and software

The single-chain structures of 18 α and 8 β integrin subunits were downloaded from the AlphaFold2 database (https://alphafold.ebi.ac.uk). Human integrin protein sequences were downloaded from the NCBI protein database (https://www.ncbi.nlm.nih.gov/protein/). Experimentally determined structures, including α_IIb_β_3_ (PDB 3FCS), α_V_β_3_ (PDB 4G1E), α_5_β_1_ (PDB 7NXD), and α_X_β_2_ (PDB 4NEH), were downloaded from protein databank https://www.rcsb.org. All integrin structures were analyzed in PyMOL version 2.5.4 (The PyMOL Molecular Graphics System, Version 2.0 Schrödinger, LLC).

### Running AlphaFold2

AlphaFold 2 Version 2.1.2 was running on the HPC Cluster at the Medical College of Wisconsin using Miniconda3 virtual environments. The AlphaFold2 downloaded reference files are located at: “/hpc/refdata/alphafold”. Customized sbatch job script was submitted for structure prediction. Typically, one GPU (--gres=gpu:1) and 100 GB memory (--mem=100gb) was requested to run AlphaFold2. The maximum job running time was set to 48 h (--time=48:00:00). To run AlphaFold2-Multimer for structure prediction of integrin heterodimers, an input fasta file containing the sequences of both integrin α and β subunits was provided. The multimer prediction function was enabled with command “--model_preset=multimer”. Full length or extracellular domain structures of integrin heterodimers without signal peptides were predicted with or without templates by setting the parameter of “--max_template_date=2000-05-14” or “-max_template_date=2023-01-01”. For integrin α_6_β_4_ structure prediction, the large cytoplasmic tail of β_4_ was truncated after KGRDV to simplify the prediction. The top ranked models were selected for further analysis.

### Comparison of AlphaFold2 predicted integrin structures

The single chain α integrin structures downloaded from AlphaFold2 database were superimposed based on the α_IIb_ calf-2 domain using the “super” command in PyMOL. The single chain β integrin structures downloaded from AlphaFold2 database were superimposed based on the β_3_ βI domain using the super command in PyMOL. The experimentally determined structures for α_IIb_ (PDB 3FCS), α_V_ (PDB 4G1E), α_5_ (PDB 7NXD), and α_X_ (PDB 4NEH) were superimposed onto the predicted corresponding structures. The experimentally determined structures for β_3_ (PDB 3FCS), β_1_ (PDB 7NXD), and β_2_ (PDB 4NEH) were superimposed onto the predicted structures accordingly. The integrin heterodimer structures predicted by AlphaFold2-multimer with or without TM-CT domains were superimposed onto the calf-2 domain of α_IIb_ in PyMOL. For structure comparison of integrin TM-CT heterodimers, the structures were superimposed based on the α_IIb_ TM domain. The aligned structures were individually oriented to position them perpendicularly to the cell membrane.

### DNA constructs

The α_5_ with C-terminal EGFP tag (α_5_-EGFP) was a gift from Rick Horwitz (Addgene plasmid #15238; http://n2t.net/addgene:15238; RRID:Addgene_15238).^46^ The α_9_ with C-terminal EGFP tag (α_9_-EGFP) was a gift from Dean Sheppard (Addgene plasmid #13600; http://n2t.net/addgene:13600; RRID:Addgene_13600). The α_3_, α_7_, and α_10_ integrins were cloned into pEGFP-N3 vector. The α_10_-N839Q mutation was generated by QuickChange mutagenesis kit (Agilent Technologies, Inc).

### Flow Cytometry Analysis of LIBS mAb binding

The HEK293T cells were grown in complete DMEM (Corning) supplemented with 10% fetal bovine serum (FBS) (Sigma-Aldrich). Cells were maintained in a 37°C incubator with 5% CO_2_. Flow cytometry analysis of integrin expression and LIBS mAb binding were as described previously.^47^ In brief, HEK293T cells were transfected with EGFP-tagged α integrin constructs plus β_1_ integrin. 48 hours post-transfection, the cells were detached, washed, and resuspended in HBSGB buffer (25 mM HEPES pH 7.4, 150 mM NaCl, 2.75 mM glucose, 0.5% BSA) containing 1 mM Ca^2+^/Mg^2+^ or 0.1 mM Ca^2+^ plus 2 mM Mn^2+^. Cells were incubated with either 9EG7 mAb (BD Biosciences) or MAR4 (BD Biosciences) for 15 mins followed by additional 15 min incubation with Alexa Fluor 647-conjugated goat anti-rat or mouse IgG. Surface binding of mAb was measured by a BD Accuri^™^ C6 (BD Biosciences). Relative surface expression of extended β_1_ integrins were normalized to total surface β_1_ integrins and plotted with Prism 9.

### Data availability

The datasets generated during and/or analyzed during the current study are available from the corresponding author on reasonable request. The predicted structures were deposited online as supplementary materials.

## Results

### The AlphaFold2 structures of 18 α and 8 β human integrins

**Figure 2.**
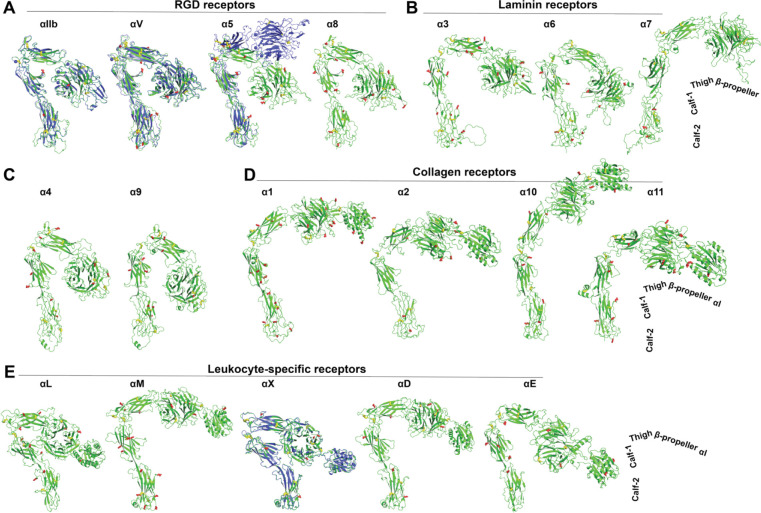
AlphaFold2 structures of the ectodomains of 18 human α integrins. The Alphafold2 structures are shown in green. The experimentally determined structures for α_IIb_ (PDB 3FCS), α_V_ (PDB 4G1E), α_5_ (PDB 7NXD), and α_X_ (PDB 4NEH) are shown in blue. The putative N-linked glycosylation sites are shown as red sticks. Disulfide bonds are yellow sticks. The structures were aligned based on the calf-2 domain and oriented perpendicularly to cell membrane.

We extracted the structures of 18 human α integrins from the AlphaFold2 protein structure database. All the structures in the database were predicted based on the full-length single-chain amino acid sequence, including the signal peptide, ectodomain, TM and CT domains. To compare the ectodomain structures of all the α integrins, we superimposed the structures based on the calf-2 domain of α_IIb_, and then the individual structures were oriented vertically to membrane normal and rotated as necessary to show the position of β-propeller domain relative to cell membrane (Fig. 2). The structures were grouped based on ligand or cell specificity. For all the structures, the correct folding of individual domains was successfully predicted (Fig. 2). The four RGD-binding α integrins all show a sharp bent conformation, with the α_IIb_ and α_V_ nearly identical to their crystal structures (Fig. 2A). However, the α_5_ AlphaFold2 structure is more bent than its half-bent cryo-EM structure (Fig. 2A). For the three laminin receptors, only α_6_ is in a sharp bent conformation as the RGD receptors, while α_3_ and α_7_ are in a half-bent conformation (Fig. 2B). The α_4_ and α_9_ integrins are also in the bent conformation as the RGD receptors (Fig. 2C). Interestingly, all the four α integrins of collagen receptors are more extended than bent, with α_10_ exists in a nearly fully extended conformation (Fig. 2D). The five leukocyte-specific α integrins also display conformational diversity, with α_L_ and α_X_ more bent than α_M_, α_D_, and α_E_ (Fig. 2E). The AlphaFold2 structure of α_X_ is nearly identical to the crystal structure (Fig. 2E).

**Figure 3.**
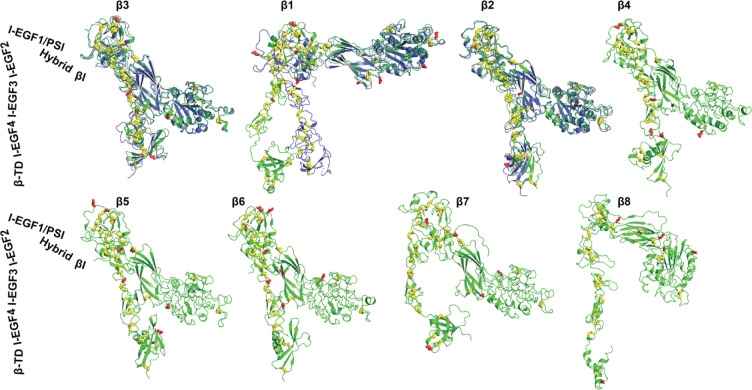
AlphaFold2 structures of 8 human β integrins. The AlphaFold2 structures are shown in green. The experimentally determined structures for β_3_ (PDB 3FCS), β_1_ (PDB 7NXD), and β_2_ (PDB 4NEH) are shown in blue. The putative N-linked glycosylation sites are shown as red sticks. Disulfide bonds are yellow sticks. The structures were aligned based on the β_3_ βI domain and oriented perpendicularly to cell membrane.

We compared the structures of 8 human β integrins that were predicted as single-chain structures in the AlphaFold2 protein structure database. The structures were superimposed based on the β_3_ βI domain and then orientated individually to position them vertically to membrane normal. As shown in Fig. 3, the correct folding of individual domains including βI, PSI, hybrid, I-EGF domains, and β-tail domain (β-TD) were accurately predicted for β_1_ to β_7_ integrins. The β-TD of β_8_ is smaller than other β integrins and its structure was incompletely predicted (Fig. 3, **β**_**8**_). The AlphaFold2 structures of β_3_, β_2_, β_4_, β_5_, β_6_, and β_7_ all assembly a bent conformation as seen in the crystal structures of β_3_ and β_2_, while the β_1_ and β_8_ structures are less bent (Fig. 3). The half-bent conformation of β_1_ AlphaFold2 structure is comparable to the cryo-EM structure (Fig. 3 **β**_**1**_).

### The domain interface where α and β integrin undergo extension

**Figure 4.**
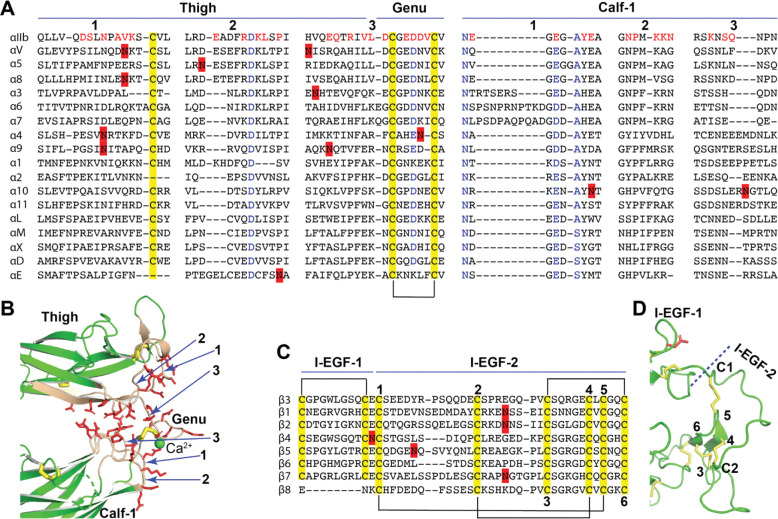
Sequence and structure of the domain interface where integrin becomes extended. (**A**) Sequence alignment of human α integrin thigh and calf-1 domain junction interface. The interfacial residues of α_IIb_ are in red. Highly conserved residues are in blue. Disulfide bonds are highlighted in yellow. The putative N-glycan sites are highlighted in red. (**B**) The interface of thigh and calf-1 of bent α_IIb_ structure. The loops at the interface are numbered in panels A and B. The interfacial residues are shown as red sticks. Disulfide bonds are yellow sticks. (**C**) Sequence alignment of human β integrin I-EGF-1 and I-EGF-2 junction. Disulfide bonds are highlighted in yellow. The putative N-glycan sites are highlighted in red. The six cysteines of I-EGF-2 are numbered. (**D**) Structure of β_3_ I-EGF-1 and I-EGF-2 junction. Disulfide bonds are yellow sticks. One N-glycan site on I-EGF-1 is shown as a red stick.

Previous structure studies revealed that the extension of α integrin happens at the interface between thigh and calf-1 domains, where located a disulfide bonded knob, denoted as genu (Fig. 1C, F). We did sequence alignment of all α integrins for the junction of thigh and calf-1 domains (Fig. 4A). The structure of α_IIb_ was used as an example to show the interface between thigh and calf-1 domains at bent conformation (Fig. 4B). The interfacial residues were shown in red in the sequence alignment (Fig. 4A) and as red sticks in the structure (Fig. 4B). Sequence alignment shows that the interfacial residues as well as the putative N-glycan sites are not well conserved (Fig. 4A). Some α integrins, such as α_V_, α_8_, α_4_, α_9_, α_10_, and α_E_ have putative N-glycan sites on the interface of either thigh or calf-1. Interestingly, the laminin receptors α_3_, α_6_, and α_7_ all have a longer interfacial loop (region 1) on calf-1 (Fig. 4A). However, there are no signature sequences that appear to prefer a bent or extended conformation.

Integrin β subunit extends at the I-EGF-1 and I-EGF-2 junction (Fig. 1C, F). Sequence alignment of 8 human β integrin at this region showed no obvious residue conservation except the classical disulfide bonds of EGF domains (Fig. 4C). In the bent conformation of β_3_ integrin (Fig. 4D), the interface between I-EGF-1 and I-EGF-2 is much smaller compared with the interface between thigh and calf-1 in α_IIb_ (Fig. 4B), and unlikely plays a major role in maintaining the bent structure. However, the length of C1-C2 loop in I-EGF-2 domain has been shown to regulate integrin extension.^48^ A landmark disulfide bond is missing in the I-EGF-1 of β_8_ (Fig. 4C), which may contribute at least in part to the distinct conformational regulation of β_8_ integrin.

### α_10_ integrin prefers an extended conformation on cell surface

**Figure 5.**
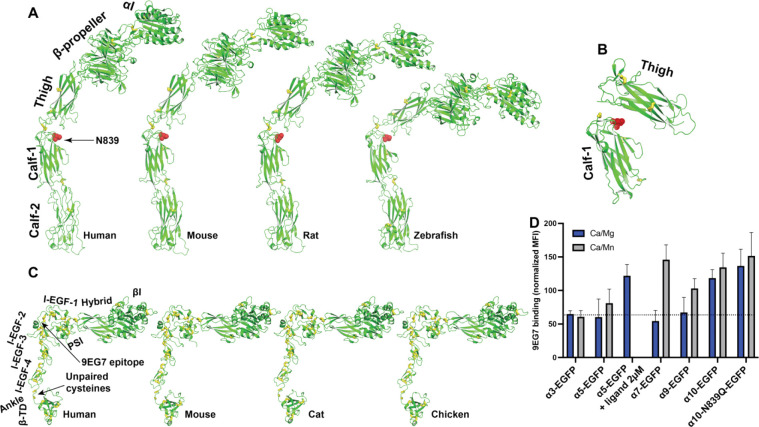
AlphaFold2 structures of α_10_ and β_1_ integrins from different species. (**A**) Structures of α_10_ integrins of human, mouse, rat, and zebrafish. The structures were aligned based on calf-2 domain and oriented perpendicularly to cell membrane. The N-glycan site at the thigh/calf-1 interface is shown as a red stick. (**B**) The relative position of α_10_ thigh and calf-1 domains in the fully bent conformation, indicating the interfacial location of the N-glycan site shown as red sticks. The thigh and calf-1 domains of α_10_ were superimposed on those of bent αIIb structure. (**C**) Structures of β_1_ integrins of human, mouse, cat, and chicken. The structures were aligned based on βI domain and oriented perpendicularly to cell membrane. (**D**) Conformation of β_1_ integrin co-expressed with selected integrin α subunit. Human integrin α subunits with C-terminal EGFP tag were co-expressed with human β_1_ in 293T cells. The binding of mAb 9EG7 or MAR4 IgG was measured by flow cytometry in the buffer containing 1 mM Ca^2+^/Mg^2+^ or 0.1 mM Ca^2+^ plus 2 mM Mn^2+^. The data are presented by the MFI of 9EG7 binding as a percentage of the MFI of MAR4 binding.

Of the 18 α integrins, the AlphaFold2 structure of α_10_ presents an extended conformation (Fig. 2D). The extended structure was also seen for α_10_ of other species, including mouse, rat, and zebrafish (Fig. 5A). We also observed a conserved putative N-glycan site (N839 in human) at the thigh/calf-1 interface of α_10_ integrin (Fig. 5A). The N-glycans at this site may interfere with the bent conformation (Fig. 5B). α_10_ integrin only forms heterodimer with β_1_ integrin. The AlphaFold2 structures of human, mouse, cat, and chicken β_1_ are all in a half-bent conformation (Fig. 5C). To assess the conformation of α_10_β_1_ on cell surface, we used mAb 9EG7, which recognizes the β_1_ I-EGF-2 epitope that is masked in the bent conformation (Fig. 5C),^49^ thus reporting β_1_ integrin extension. α_3_, α_5_, α_7_, and α_9_ integrins were used for comparison. The α integrins were expressed as EGFP-fusion proteins along with β_1_ in HEK293T cells. Flow cytometry analysis showed that, under the physiological metal ion condition (1 mM Ca^2+^/Mg^2+^), α_10_-EGFP/β_1_ cells bound more 9EG7 than other integrins (Fig. 5D). By contrast, α_7_-EGFP/β_1_ and α_9_-EGFP/β_1_ cells only bound more 9EG7 in the activating condition (0.1 mM Ca^2+^/2 mM Mn^2+^), while α_3_-EGFP/β_1_ had no response to Mn^2+^ (Fig. 5D). α_5_-EGFP/β_1_ also didn’t response well to Mn^2+^ for 9EG7 binding compared with α_7_ and α_9_, but the RGD-like compound, MK-0429, greatly induced 9EG7 binding (Fig. 5D). α_10_-EGFP/β_1_ bound 9EG7 to a high level at both Mg^2+^ and Mn^2+^ conditions. However, mutating the putative N-glycan site (α_10_-N839Q) didn’t affect 9EG7 binding (Fig. 5D). These data suggest that α_10_ integrin exists in a constitutively extended conformation on cell surface.

### The integrin α/β heterodimer structures predicted by AlphaFold2-multimer

**Figure 6.**
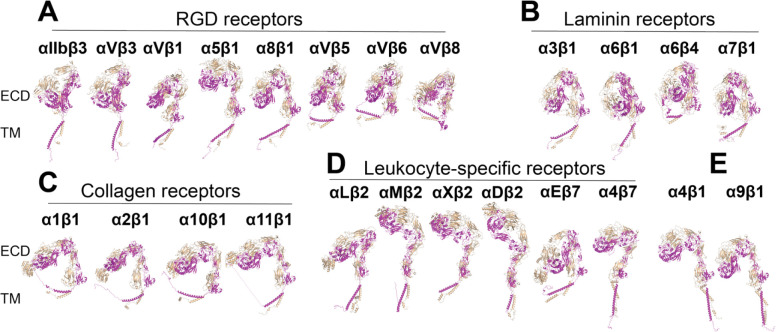
Structures of 24 human integrins predicted by AlphaFold2-multimer. (**A-E**) The full-length integrin structures were calculated by AlphaFold2-multimer with the max_template_date of 2000. Structures were superimposed onto the calf-2 domain of α_IIb_ subunit and shown in the same orientation. The α and β subunits are shown in wheat and magenta, respectively.

The integrin structures in AlphaFold2 database are only for single-chain α and β subunits. We utilized the AlphaFold2-multimer module to predict the α/β heterodimer structures of all 24 human integrins. To avoid any potential model bias from known integrin structures, we set the template search date at the year 2000, prior to which no integrin structures had been reported. All 24 integrin structures were successfully predicted by AlphaFold2-multimer (Fig. 6). All the structures were superimposed onto the α_IIb_ calf-2 domain and then orientated individually to position the ectodomains vertically to membrane normal and grouped based on ligand or cell specificity (Fig. 6). Overall, the inter-subunit interfaces such as that of α β-propellor and β βI domains were correctly predicted. The α_IIb_β_3_ and α_V_β_3_ ectodomain structures are very close to the crystal structures with Cα RMSD about 2 Å. All the RGD receptors assembled a bent conformation, except α_5_β_1_ is half-bent (Fig. 6A). Similarly, all the laminin receptors are also bent (Fig. 6B). The four collogen receptors all show a half-bent structure, including α_10_β_1_ (Fig. 6C). α_10_β_1_ was suggested to be more extended based on 9EG7 mAb binding (Fig. 5D). Among the leukocyte-specific integrins, only α_L_β_2_ and α_E_β_7_ are sharp bent, while α_M_β_2_, α_X_β_2_, α_D_β_2_, and α_E_β_7_ are more extended (Fig. 6D). The α_4_β_1_ and α_9_β_1_ are in half bent conformation (Fig. 6E). The relative orientation of TM domains to the cell membrane are incorrectly predicted for most of the structures.

**Figure 7.**
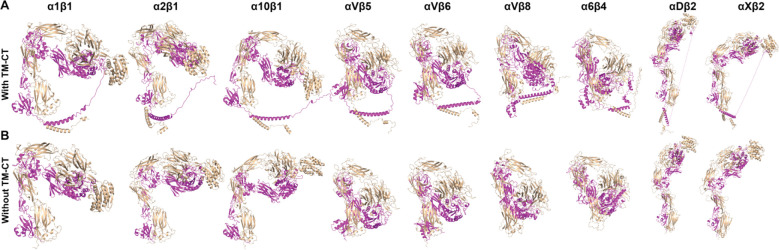
Comparison of structures predicted by AlphaFold2-multimer with and without transmembrane and cytoplasmic domains. (**A**) The full-length structures of some integrins calculated by Alphafold2-multimer show artificial contacts between ectodomain and TM-CT domains. (**B**) The structures in panel A were re-calculated in the absence of TM-CT domains. The structures were superimposed based on the calf-2 domain of α subunit and shown in the same orientation. The α and β subunits are shown in wheat and magenta, respectively.

Since 9 of the integrin structures predicted by AlphaFold2-multimer show interactions between TM-CT and ectodomains (Fig. 7A), we asked if such artificial interactions affect the overall integrin structure prediction. We re-calculated the 9 integrin structures without TM-CT domains using AlphaFold2-multimer. The structures show essentially the same conformations as those with TM-CT domains (Fig. 7B), suggesting that the folding of ectodomain and TM-CT does not influence each other during the structure calculation by AlphaFold2-multimer.

**Figure 8.**
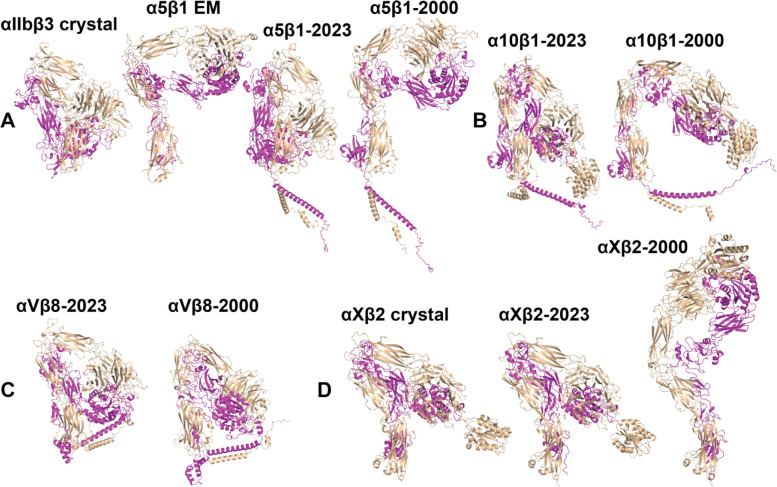
Comparison of selected structures predicted by AlphaFold2-multimer with and without enabling the option of using known structures as templates. (**A**) The full-length α_5_β_1_ structures were calculated by AlphaFold2-multimer with the max_template_date of 2023 or 2000. The crystal structure of α_IIb_β_3_ (PDB 3FCS) and cryo-EM structure of α_5_β_1_ (PDB 7NXD) were shown for comparison. (**B**) AlphaFold2 structures of full-length α_10_β_1_ with the max_template_date of 2023 or 2000. (**C**) AlphaFold2 structures of full-length α_V_β_8_. (**D**) AlphaFold2 structures of ectodomain of α_X_β_2_. The crystal structure of α_X_β_2_ (PDB 4NEH) was shown for comparison.

Next, we investigated whether enabling the template option for AlphaFold2-multimer had any impact on the calculation of integrin structures. The α_5_β_1_, α_10_β_1_, α_V_β_8_, and α_X_β_2_ integrins were selected for the test. When setting the template searching date at the year of 2023, all four integrin structures calculated by AlphaFold2-multimer showed a sharp bent conformation (Fig. 8A–D), which closely resembled the crystal structure of bent α_IIb_β_3_ (Fig. 8A). Notably, the AlphaFold2-multimer predicted α_5_β_1_ structure with a template search date set at 2023 is much more bent than the one with a search date set at 2000, which closely resembles the α_5_β_1_ cryo-EM structure (Fig. 8A). Similarly, the α_10_β_1_-2023 is bent comparing with half bent α_10_β_1_-2000 structure (Fig. 8B). However, both α_V_β_8_-2023 and α_V_β_8_-2000 structures are bent to a similar level (Fig. 8C). In sharp contrast, the α_X_β_2_-2023 structure shows a conformation resembling the bent α_X_β_2_ crystal structure, while the α_X_β_2_-2000 structure is extended (Fig. 8D). These findings suggest that the inclusion of template structures can significantly affect the structure prediction outcomes of AlphaFold2-multimer.

### Structures of integrin TM-CT domains

**Figure 9.**
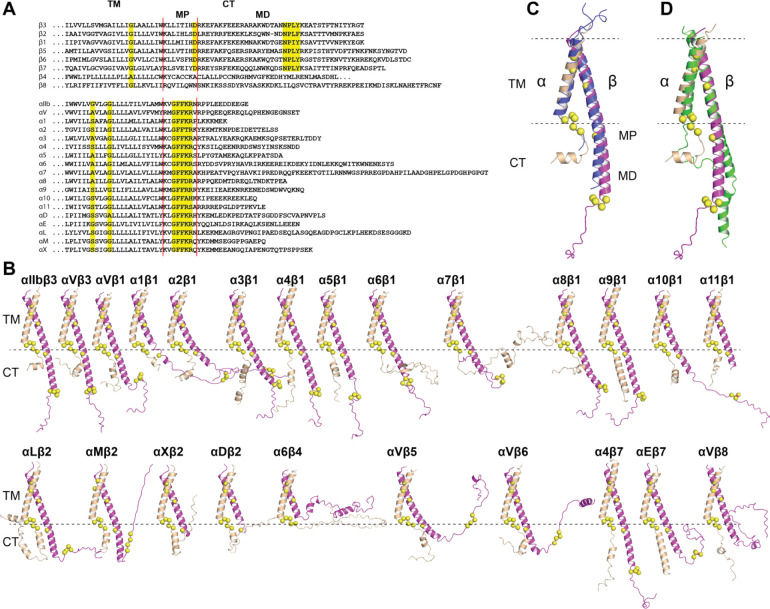
Structures of integrin transmembrane and cytoplasmic domains predicted by AlphaFold2-multimer. (**A**) Sequence alignment of human β and α integrin TM and CT domains. The conserved residues are highlighted in yellow. The boundaries of membrane proximal (MP) and membrane distal (MD) regions of CT domain are indicated. (**B**) AlphaFold2 structures of 24 human integrin TM-CT domains. The structures were superimposed onto the TM domain of α_IIb_ subunit and shown in the same orientation. The conserved residues highlighted in panel A are shown as yellow Cα spheres. (**C**) Superimposition of AlphaFold2-predicted α_IIb_β_3_ TM-CT structure (in wheat and magenta) on the heterodimeric structure of α_IIb_β_3_ TM-CT structure determined by disulfide crosslinking and Rosetta modeling (in blue). The α and β subunits are shown in wheat and magenta, respectively. (**D**) Superimposition of α_IIb_β_3_ TM-CT structure predicted without (green) and with ectodomain (wheat and magenta).

Despite the relative simplicity of the sequence and structure of integrin TM and CT domains, only the structure of the α_IIb_β_3_ TM-CT heterodimer has been experimentally determined to date. Sequence alignment of the TM-CT domains from 8 β and 18 α human integrins reveals conservative features at the TM, membrane-proximal (MP), and membrane-distal (MD) regions (Fig. 9A). We analyzed the 24 integrin TM-CT structures calculated by AlphaFold2-multimer (Fig. 9B). Structure alignment reveals a high degree of structural similarity for the heterodimers at TM domain, highlighting the conserved α GXXXG motif and β small G/A residue on α/β interface (Fig. 9B). The conserved GFFKR motifs in the CT MP regions of 18 α integrins all adopt a reverse turn conformation, while the β CT MP regions, except for β_4_ and β_8_ integrins, all display an α-helical structure extended from the TM region, with the conserved Asp residue located on α/β interface (Fig. 9B). The β CT Asp residue is proximal to the Arg residue in the α GFFKR motif (Fig. 9B), which was proposed to form a salt bridge interaction.^50^ The CT MD regions of both α and β subunits exhibit diverse disordered conformations, including the conserved NPXY motif that binds talin, as shown in Figure 9B. Despite not allowing any integrin TM-CT structure templates during the AlphaFold2-multimer calculation, the predicted structure of α_IIb_β_3_ TM-CT closely resembles the experimentally determined structure (Fig. 9C). We also generated a prediction for the α_IIb_β_3_ TM-CT structure in the absence of the ectodomain, which showed a similar TM interface to that predicted with the ectodomain present (Fig. 9D). These results suggest that AlphaFold2-multimer is capable of accurately predicting integrin TM structures.

## Discussion

Sequence alignment analysis revealed the averaged sequence identity was 30–40% among the 8 β integrins, and 20–40% among the 18 α integrins.^38^ For individual integrin domains, such as the βI domain, the sequence identity can be up to over 60%.^38^ Since AlphaFold2 incorporates amino acid sequence, multiple sequence alignments and homologous structures in its structure calculation,^44^ the high sequence identity observed among integrin domains may facilitate the accurate prediction of integrin domain structures for most of the family members. This is demonstrated by the comparison of predicted structures with the experimental structures of α_IIb_β_3_, α_V_β_3_, and α_X_β_2_, which show a high degree of structural similarity. Thus, the predicted integrin domain structures can be utilized with a high level of confidence.

Our sequence and structure analysis of the inter-domain interface in bent integrin conformations did not reveal any signature sequences that clearly favor either a bent or extended conformation for both α and β subunits. Despite using bent α_IIb_, α_V_, and α_X_ structures in the PDB as part of its training sets, AlphaFold2 was able to predict a diverse range of conformations for the 18 single-chain α structures, ranging from sharp bent to almost fully extended structures, as seen in the case of α_10_. In contrast, the eight single-chain β structures predicted by AlphaFold2 were mostly bent. Also, AlphaFold2-multimer predicated both bent and extended conformations of α/β heterodimers. While our functional assay suggested that α_10_β_1_ may adopt an extended conformation on the cell surface, consistent with the predicted single-chain α_10_ structure, the heterodimer structure of α_10_β_1_ predicted by AlphaFold2-multimer does not exhibit such a conformation. Since integrins can adopt multiple conformational states, the predicted structures by AlphaFold2 may reflect such conformational diversity. We observed that the AlphaFold2-multimer prediction may be influenced by homologous structures, which could potentially introduce bias in predicting the overall conformation of integrins. AlphaFold2-multimer has demonstrated remarkable success in accurately predicting the structures of protein complexes, including those with transient interactions, multiple subunits, and large interfaces.^51, 52^ Here, we showed that the AlphaFold2-multimer algorithm can successfully predict large complexes of integrin heterodimer structures.

Although AlphaFold2 has its limitations as many other protein structure prediction programs, its ability to predict integrin domain structures, inter- and intra-domain interfaces, and overall domain organization with an acceptable level of accuracy makes the predicted structures highly useful for designing both functional and structural studies. These structures can be used to analyze interesting N-glycan sites, functional mutations, antibody epitopes, and design constructs for integrin expression and purification. Additionally, the structures can be used for molecular dynamics simulations, ligand docking, and interpreting some functional data. However, caution is needed when using these predicted structures without experimental validation.
